# Effectiveness and implementation of an obesity prevention intervention: the HeLP-her Rural cluster randomised controlled trial

**DOI:** 10.1186/1471-2458-14-608

**Published:** 2014-06-16

**Authors:** Catherine B Lombard, Cheryce L Harrison, Samantha L Kozica, Sophia Zoungas, Catherine Keating, Helena J Teede

**Affiliations:** 1 Monash Centre for Health Research and Implementation (MCHRI), School of Public Health and Preventive Medicine, Monash University, Melbourne, Australia; 2 Diabetes and Vascular Medicine Unit, Monash Health, Melbourne, Australia; 3 Deakin Health Economics, Deakin University, Melbourne, Australia

**Keywords:** Obesity, Prevention, Lifestyle, Women, Rural, Implementation, Translation, Population, RE-AIM

## Abstract

**Background:**

To impact on the obesity epidemic, interventions that prevent weight gain across populations are urgently needed. However, even the most efficacious interventions will have little impact on obesity prevention unless they are successfully implemented in diverse populations and settings. Implementation research takes isolated efficacy studies into practice and policy and is particularly important in obesity prevention where there is an urgent need to accelerate the evidence to practice cycle. Despite the recognised need, few obesity prevention interventions have been implemented in real life settings and to our knowledge rarely target rural communities.

**Methods:**

Here we describe the rationale, design and implementation of a Healthy Lifestyle Program for women living in small rural communities (HeLP-her Rural). The primary goal of HeLP-her Rural is to prevent weight gain using a low intensity, self-management intervention. Six hundred women from 42 small rural communities in Australia will be randomised as clusters (n-21 control towns and n = 21 intervention towns). A pragmatic randomised controlled trial methodology will test efficacy and a comprehensive mixed methods community evaluation and cost analysis will inform effectiveness and implementation of this novel prevention program.

**Discussion:**

Implementing population interventions to prevent obesity is complex, costly and challenging. To address these barriers, evidence based interventions need to move beyond isolated efficacy trials and report outcomes related to effectiveness and implementation. Large pragmatic trials provide an opportunity to inform both effectiveness and implementation leading to potential for greater impact at the population level. Pragmatic trials should incorporate both effectiveness and implementation outcomes and a multidimensional methodology to inform scale-up to population level. The learnings from this trial will impact on the design and implementation of population obesity prevention strategies nationally and internationally.

**Trial registration:**

ANZ clinical trial registry ACTRN12612000115831. Date of registration 24/01/2012

## Background

Longitudinal data indicates mean body mass index (BMI) is increasing across all population groups. In Australia, approximately one-fifth of those who are normal weight or overweight progress to a higher weight category within 5 years [1,2]. For most people weight gain increases gradually over several decades, and is estimated to be on average 600-800 g per year [3,4]. Weight gain, even in those who are a healthy weight, is associated with an increased risk of chronic disease [5]. This supports the need for interventions to prevent weight gain in those who are currently within the healthy weight range, as well as those who are overweight or obese. Population subgroups at higher risk for rapid weight gain include young women [3] particularly those living in rural communities [6,7]. Despite the direct links between weight gain and increased risk of obesity related diseases, few interventions have specifically targeted the prevention of weight gain or women living in rural communities [6,8,9].

Prevention of weight gain is feasible, requiring only minor adjustments to lifestyle behaviours [4,10]. Our team was one of the first to demonstrate prevention of weight gain in a clustered randomised controlled trial (RCT) of 250 women with a mean age 39 years. Compared to standard health information alone, the Healthy Lifestyle Program (HeLP-her) a low intensity, self-management intervention, significantly reduced weight gain over one year in an urban population of women [4]. Following the success of HeLP-her trial in an urban population we now seek to expand the evidence for this program to include outcomes relevant to effectiveness and implementation.

A gap in research that translates evidence-based interventions from isolated efficacy trials toward broad effective population strategies is evident [11,12]. Implementation research seeks to address this gap by generating an understanding of how interventions work within real world conditions [11]. The hybrid effectiveness-implementation model retains essential features of efficacy trials (randomisation and control groups) while addressing issues important in implementation (reach, diversity of settings, satisfaction and cost) [13]. Applying this effectiveness–implementation model to obesity prevention in the community will answer important questions related to successful implementation and expedite the successful implementation of interventions into practice.

The aim of the HeLP-her Rural study is to evaluate the efficacy, effectiveness, impact and cost of implementing the HeLP-her program in small rural communities in Australia. The hybrid effectiveness–implementation design features a cluster RCT, a mixed methods community evaluation and an economic analysis to inform scale-up into diverse populations and add to current knowledge on implementing prevention interventions in general.

## Methods

### Study design

Help-her Rural is a community based, cluster RCT design. The RCT involves an active intervention phase (year 1) and an observational phase (year 2) concurrent with a comprehensive community evaluation (participants and stakeholders) and economic evaluation. The primary aim of this trial is to compare the effect of a low intensity intervention (HeLP-her Rural), with an information only control on weight gain in reproductive aged women living in small rural communities in Australia. The secondary aims are to improve health behaviours including diet quality and regular participation in physical activity; to assess the impact of HeLP-her Rural on participant engagement, health behaviour, attitudes and knowledge; to assess stakeholder experiences; and assess the cost-effectiveness of the program. Phase 1 includes a one-year, low intensity intervention using face-to-face, telephone and text messaging. Phase 2 is an observation year with no active intervention for either intervention or control groups. The study will be conducted in accordance with the Consolidated Standards of Reporting Trials (CONSORT) guidelines.

The Monash Health Human Research and Ethics Committee has approved the study, project No.12034B.

### Selecting communities

Victoria is a southern State of Australia with 34 Shires of defined geographical boundaries. Population statistics and socioeconomic data are readily available for each. Twelve of these Shires will be excluded from recruiting because of potential confounding related to their participation in a Government funded preventative health campaign. Within the remaining 22 Shires, all small rural towns with populations of between 1500–10,000 persons, located within a radius of between 100 km and 400 km from Melbourne Central Business District (CBD), will be eligible for randomisation. Town selection criteria is based on size and distance from the CBD as these factors are likely to impact on availability and access to health and community services readily available in larger regional towns. Based on these criteria, 44 towns will be eligible for randomisation.

### Sample size calculations

In the calculation of sample sizes for the primary outcome (weight change over 12 months) adjustments will be made for the cluster design. The variance inflation factor (VIF) used to achieve this is determined from the average cluster size and the Intra-Cluster Correlation (ICC). We have powered the trial to detect a difference of 1.0 kg in weight between groups at 12 months based on our previous trial [4] and estimated population weight gain [3]. Assuming a significance level of 90% for a two-sided test, 196 women per group will be required to detect the absolute difference in weight with 80% power. The actual ICC calculated in the previous HeLP-her was −0.02 using Generalised Estimating Equations (GEE) [14], and despite the negative value is assumed to be equivalent to zero. However, we are assuming some clustering in this setting although small, and as there is little published data on which to base an estimate in rural community groups, we have assumed an ICC =0.02, cluster size m = 15, and a VIF 1.28 (1 + (m-1)*0.02)*195). Therefore, 250 women per group will be required and allowing for 20% attrition in participants over 12 months we plan to recruit 600 women in 40 clusters of 15 women. Because of the complexity and cost of the RCT we plan to oversample by 2 towns and randomise 42 towns using computer generated numbers, thereby allowing for any inadvertent recruiting issues.

### Inclusion and exclusion criteria

We aim to be as inclusive of the community as possible and hence have minimal exclusion criteria. We will recruit adult women (18–50 years) who plan to reside in the community for at least the next 2 years. Women will be excluded if they have a serious physical or psychological condition that might affect their ability to complete outcome measures or participate fully, are pregnant or breast feeding, taking weight control medications or who have had bariatric surgery. To be inclusive of all community members, body mass index (BMI) is not used as an inclusion or exclusion criteria.

### Program theory and implementation strategy

The HeLP-her Rural intervention is based on our previous successfully delivered HeLP-her intervention [15], which was delivered to women living in urban communities. The intervention has been adapted to address potential barriers to implementation in a rural setting. Barriers for both delivery and participation in health programs in a rural setting include travel distance and time required to attend sessions. To address potential participation barriers we will substitute some face to face meetings with remote delivery, be flexible in meeting times ensuring convenience, hold sessions in local settings such as schools, pre-schools and community centres, and allow young children to attend.

The following principles will guide the implementation and evaluation of HeLP-her Rural in this community setting. These principles are based on our previous work [15], similar interventions [16] as well as national and international guidelines and frameworks [17].

The intervention will

1. Align with national and international health priorities

2. Be theory driven

3. Include multi-sectoral communication and engagement

4. Be adaptable to local context

5. Aim to recruit a representative sample of the target population

6. Address barriers to participation and be a low burden on participants to maximise reach

7. Align with and support, local programs and strategies thereby adding value to local prevention interventions

8. Provide the highest quality evidence available

9. Be pragmatic with recruitment and delivery as close to real life as practicable

10. Assess reach, effectiveness, adoption and implementation via a comprehensive community evaluation

11. Be low intensity to ensure low cost and sustainability

#### Program theory

The HeLP-her intervention is based on the self-determination theory (SDT) with motivational interviewing (MI) [18] and self-management principles providing the basis for content, resources and any personal contact by the program facilitator. In keeping with the SDT, HeLP-her aims for participants to consider their own personal needs, skills, priorities and goals. Application of MI aims to move participants to choosing and then valuing the outcomes of their behaviour. The content is non-prescriptive, instead providing general health messages and a focus on small achievable changes to enhance self-efficacy and sustainability of behaviour change. We adapted the taxonomy developed by Haase et al. to identify SDT components in the HeLP-her Rural program [19] (Table 1).

**Table 1:** SDT components of HeLP-her Rural

**SDT components**	**Content activities**
**Competence**	Describe the pattern of weight gain in women
	Explore value and benefits of changing behaviour
	Introduce general health messages based on physical activity and healthy eating behaviours
	Identify personal barriers and enablers to behaviour change.
	Learn behavioural skills such as goal setting, problem solving, relapse prevention, self-monitoring
**Autonomy**	Explore beliefs and value of changing personal behaviours, identify personal priorities
	Explore small achievable steps and realistic expectations
	Explore choices and identify personal priorities, goals and action plans
	Explore where to seek further information and support
	Learn how to monitor and review progress
	Explore ambivalence and barriers
**Issues relating to people, environment and context**	Support links within the community
	Engage support from friends and family

### Program Implementation strategy

A comprehensive communication plan and engagement framework has been developed in order to ensure efficient multilevel engagement across communities (Table 2).

**Table 2:** Community communication and engagement strategy

**Stakeholder group**	**Method of contact**	**Purpose**
Government Departments	Letter to provide general program information and point of contact	Government departments can be powerful allies if the program aims align with government targets. They have knowledge of communities and can connect to local stakeholders.
Local health sector and local government	Telephone call followed by email of program information and flyers.	Local health workers and local government workers have good local knowledge of their own population, health issues, local champions and potential collaborators. They can assist recruitment and reach through and their databases and contacts and support with venues.
	Regular program updates	
Private sector and	Telephone call and follow up email.	Local champions have good communication skills and support local recruitment. They distribute flyers and encourage their networks to participate.
Local champions		
Community sector, sports clubs, women’s groups, neighbourhood houses	Telephone and send program information and flyers.	Local organisations provide opportunity for recruitment, distribute flyers and identify potential links with local programs.
All local print and radio media outlets identified	A media release sent, followed up by a telephone call to the editor/producer.	We focus on local newspapers, offer photo opportunities and interviews to increase awareness and recruitment.
School Principals, Kindergarten Directors	Telephone calls, meetings, and program information.	Schools, kindergartens, childcare give direct access to participants. Principal support can validate the program. They provide practical support by distributing and promoting the program, providing space and refreshments.

#### Recruiting strategies

Simple recruiting methods will include in-person, letters and flyers, media and ‘local champions’. Based on our previous experience and the age of our target group, a primary source of recruitment will be day-care, pre-school and primary schools. The recruitment strategy in the 6 weeks prior to intervention commencement includes: 6 weeks prior communicate with primary school principles, pre-school and day care centres and engage their support; 4 weeks prior distribute letters and flyers to all families in primary schools and pre-schools; 4 weeks prior send a media release to all local media outlets; 2 weeks prior researchers visit each town to meet with local stakeholders and recruit in person. From prior experience we believe women respond better to program recruitment when there is some personal interaction. Women will be invited to express interest in person, via text message, telephone, and email or by returning a form to a collection point. Interested women will be screened by telephone and if eligible sent information packs including informed consent and baseline questionnaires prior to program commencement.

#### Facilitator training

Facilitators who have completed program specific training will deliver the intervention, including phone coaching. Facilitators will be required to have a tertiary qualification in health sciences and undergo one day training. Facilitators need excellent interpersonal skills, a sound knowledge of evidence based practice, an understanding of health, health behaviour, nutrition, physical activity and some knowledge and experience in communicating with a variety of sectors. Program specific training includes both theory and practical components and motivational interviewing techniques.

#### Program resources

Program resources (peer reviewed and consumer tested) include an interactive manual containing assessments, health information, personal stories, as well as tools to self-monitor and assess progress that aim to develop and improve skills in self-management. Additionally, intervention participants will have access to a password protected website containing information on the trial, health information, notification of session times and team information and contact details.

#### Program fidelity and adherence

Program fidelity will be maintained by self-assessment using a checklist completed by the facilitator following delivery and reviewed by the research assistant who attended the same session to reduce potential reporting bias. Participant adherence is checked at 12 weeks in the phone coaching session where participants will be asked if they have completed the manual activities. Participants will work through missing action plans or manual activities with facilitators to ensure the intervention is received as intended.

### The intervention

#### Intervention components and delivery overview (Figure 1)

**Figure 1:**
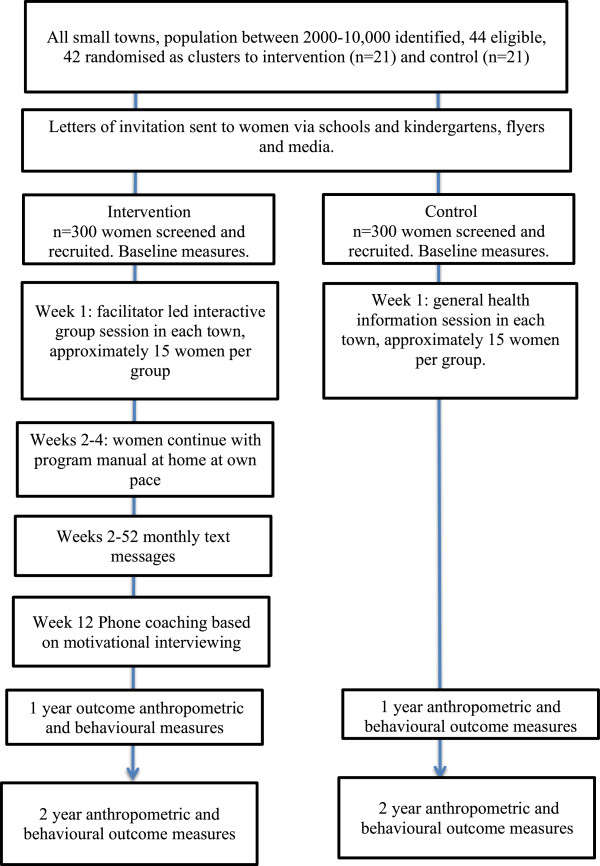
HeLP-her Rural intervention flow chart.

Week1: Groups of approximately 15 participants will attend a single group session held at a local community centre such as a primary school, community hall, pre-school, club or health service. Following completion of baseline measures and questionnaires a trained facilitator will deliver the skill-based activities in small groups. Participants will complete section 1 during the delivery session, with activities and discussion led by the facilitator. Following the session, women will complete the manual activities in their own time over the next 2–4 weeks. Women will be given access to the program website.

Weeks 2–12: Text messages will be sent to mobile phones weekly in the first 4 weeks, then one every 4 weeks to remind participants of intervention behavioural messages and to complete manual activities.

Week 12: One 20-minute telephone coaching call, based on motivational interviewing will be delivered to all intervention participants. The telephone call will also check compliance and ensure program activities have been completed

Weeks 12–52: On-going support will be provided by monthly mobile phone text messages sent to mobile phones to remind participants of intervention behaviour messages.

### The intervention detail

#### Knowledge generation

The group session aims to generate a shared understanding and knowledge of health, healthy eating behaviours and physical activity behaviours. Themes within the group session include weight gain in women, health impacts and statistics to allow women to realistically place themselves within the whole population to ensure they feel they are not alone and the behaviours they struggle with are common.

Simple messages regarding eating and physical activity behaviours give women an achievable context in which to begin to formulate their own priorities. The program is deliberately not prescriptive and will focus on the behaviours around physical activity and eating rather than when or how to perform these behaviours.

#### Behavioural skills

All program activities focus on increasing awareness through personal stories, identifying personal barriers and enablers and developing goals through activities and self-assessments designed to enhance intrinsic motivation and increase self-confidence. We focus on developing realistic and achievable goals by teaching participants to break large goals into small steps. We re-focus goals toward intrinsic motivation by using activities that allow women to identify what lifestyle behaviours are managed positively, what behaviours they wish to improve and thereafter prioritising these to produce a behavioural goal. Action plans are used as a way of summarising thoughts and documenting them so they can be monitored, reviewed and improved.

#### Local adaptation

The intervention design is flexible and will allow facilitators to tailor the program with participant needs and the interactive session allows participants to spend more time on topics of interest to them. For example, adaptations to the personal stories and practical examples and group discussion topics may vary. The delivery location might be a school, pre-school or community centre depending on resources available.

#### Support social connectedness

During the group session women will be encouraged to support each other and join compatible local programs. Providing links with local programs has reciprocal advantages for both participants and the community by boosting local participation. We will communicate regularly with women via text messages and a website to maintain links and remind women of program messages and behavioural skills.

### Control group

Participants attend in groups of approximately 15 women and following completion of baseline questionnaires and measures, receive a single one hour non-interactive women’s health information session focusing on a general women’s health. Content will be based on readily available Australian Dietary Guidelines and Physical Activity Guidelines for adults [20,21] and associated pamphlets provided. No individual advice on weight will be provided or further support or contact for the duration of the 2-year program.

### Outcome measurements

The primary outcome of HeLP-her Rural is the difference in weight gain between the control and intervention communities at 1 year and 2 years. Weight, height and waist and hip circumference measurements will be collected by trained researchers (unblinded) at baseline, 1 year and 2 years. Blinding is not generally feasible in pragmatic community wide research. Measurements will be taken in light clothing, without shoes and with an empty bladder. All participants will be weighed to the nearest 0.1 kg on calibrated electronic scales (Tanita WB110AZ). Height will be measured using portable stadiometer to the nearest 0.1 cm. Dietary intake will be assessed using the Cancer Council Australia Food Frequency Questionnaire [22] and physical activity, via the International Physical Activity Questionnaire (long version). An adapted physical activity and dietary self-management questionnaire [23] and PANSE self-efficacy endpoints via questionnaire [24] will be collected.

### Community evaluation

A novel component of the methodology is a comprehensive community evaluation**.** The evaluation strategy forms a sub-study nested within the larger HeLP-her Rural RCT, focusing on translation and transferability into real-world settings. The evaluation will include a process evaluation to assess program implementation including reach, fidelity, context, quality and dose delivered to and received by participants. A summative evaluation will also be conducted to draw conclusions regarding the impacts and benefits of the program. These aspects will be measured using a mixed-methods approach including semi-structured interviews with participants, stakeholders and researchers, as well as developed program specific checklists. We will use elements of the RE-AIM framework to inform the community evaluation [25].

### Economic evaluation

An incremental cost-effectiveness analysis will determine whether the HeLP-her Rural intervention represents “value-for-money” measured against the control intervention. Both a trial-based evaluation and a modelled lifetime evaluation will be conducted from a ‘Government healthcare as 3rd party payer’ perspective.

The *Trial-Based Evaluation* will analyse costs and outcomes observed during the active intervention. Detailed pathway analysis will be used to specify all activities undertaken as part of the intervention in order to measure costs of associated resource use. Costs will include “intervention costs” (e.g. Groups, facilitation, website maintenance, text messaging). Unit costs will be drawn from best available sources for the 2012 reference year. Outcomes as observed in the RCT will be combined with the cost data to report results expressed as the ‘the $ cost per kilogram gain prevented’.

The *Modelled Economic Evaluation* will build on the trial-based evaluation by extrapolating costs and outcomes over the lifetime of the trial participants. A Markov model will be developed to estimate the lifetime costs and quality-adjusted life years (QALYs) of patients. The analysis will assume that there are no further intervention costs after the one-year active intervention. Assumptions regarding the maintenance of the intervention effect (weight gain prevention for the intervention group) will be based on sustainability of effect observed during the post-trial observation period (data collected at 2 years) combined with published literature regarding the long-term sustainability of behaviour change interventions. QALYs will be estimated based on body mass index-specific quality of life weights and mortality rates. The cost side of the analysis will be expanded to include cost “offsets” (i.e. the costs to treat overweight/obesity-related morbidity and complications). The incremental cost-effectiveness ratio will be reported as the ‘$ cost per QALY saved’ and compared with the commonly used benchmark of ‘value-for-money’ in Australia (e.g. $50,000 per QALY saved). Standard discounting will be applied to both costs and outcomes and simulation-modelling techniques will be utilised to calculate 95% uncertainty intervals. Supplementary modelling will extrapolate results for all women in the target group at the national level.

## Discussion

Interventions to prevent weight gain are rare with recent systematic reviews identifying only nine interventions. Few interventions go beyond exploring efficacy outcomes. [8,9]. The HeLP-her Rural trial uses a novel small behaviour change approach in order to prevent weight gain in women living in rural communities. We use simple, recruitment strategies to recruit a representative sample of women and deliver a low intensity self-management intervention by trained facilitators. The overarching program principles are to develop a low cost, effective program, maximise reach, deliver locally and build on the social connections that already exist in these communities.

The RE-AIM framework, an established health promotion planning and evaluation framework, will be used along with efficacy outcomes, to address issues related to reach, effectiveness (including generalisability) and sustainability of the program and inform eventual scale up for population roll out. For example our broad inclusion criteria, communication with multiple sectors and alignment with local programs, aims to maximise reach into the target group to engage and retain a representative sample of women. Additionally, the value of programs such as HeLP-her is not just in the impact it has on program attendees but the reach beyond program participation. Aligning HeLP-her with these local needs and existing programs and communicating this as ‘value adding, not ‘replicating’ is important for cross sector engagement and maximising reach. The same strategy addresses program adoption (uptake at organisational level) by connecting with and supporting local stakeholders, raising awareness and demonstrating low cost delivery, feasibility and program effectiveness. Pragmatic trials should not be static but be adaptable to local context [11]. For example our training program will ensure consistency in the delivery of the core program elements, but within the training program opportunities for adaption around these core elements is discussed, such as selecting case studies and discussion topics to reflect interests of the group. Additionally, intervention intensity (the burden on participants and facilitators) and cost to deliver the program, impact on the potential for scale up. We believe our small changes, non-prescriptive approach, recruiting strategy and flexible delivery will ensure good recruitment and high retention rates for this study. An observation year follows the active intervention and will answer questions related to maintenance of behaviours long term.

The HeLP-her Rural trial has implications for the implementation of preventative health strategies broadly. By using quantitative and qualitative methodologies we will answer important questions related to engagement, behaviour change and program sustainability at both participant and stakeholder level. Our comprehensive evaluation will examine program participation and an in-depth analysis of participant responses will help determine why women did or did not participate; the extent of behaviour change; the facilitators of behaviour change; and also investigate the natural history of program continuation or decline. Interviews with stakeholders will inform future development of obesity prevention programs that are feasible, that the community will adopt and will do this within a context of a low intensity, self-management program.

This intervention is novel due to the focus on prevention in a rural cohort of young women, the low intensity delivery and use of new technology such as text messages and phone coaching in delivery. The strengths of this trial are the inclusion objective weight measures as well as a range of behavioural outcomes, community evaluation and economic analysis supported by a robust cluster RCT design. This is one of the largest prevention focused RCTs in women and will provide much needed evidence on the efficacy, effectiveness and successful implementation of prevention interventions generally.

## Conclusions

This hybrid effectiveness-implementation trial with integrated evaluation and cost effectiveness is novel as it targets the prevention of weight gain. The trial is robustly designed and will ensure research investment yields population health benefits.

## Competing interests

The authors declare that they have no competing interests.

## Authors’ contributions

CBL, HJT, conceptualised the trial. CBL, HJT, SZ provided intellectual input into the trial design and methodology. CBL, CLH, SLK implemented the trial, delivered the intervention and collected data. SLK with HJT, CBL and CH designed the evaluation methodology and data collection. CK with CBL and CLH designed the economic evaluation methodology. CBL drafted the manuscript and all authors contributed to, reviewed and approved the manuscript.

## Pre-publication history

The pre-publication history for this paper can be accessed here:

http://www.biomedcentral.com/1471-2458/14/608/prepub
